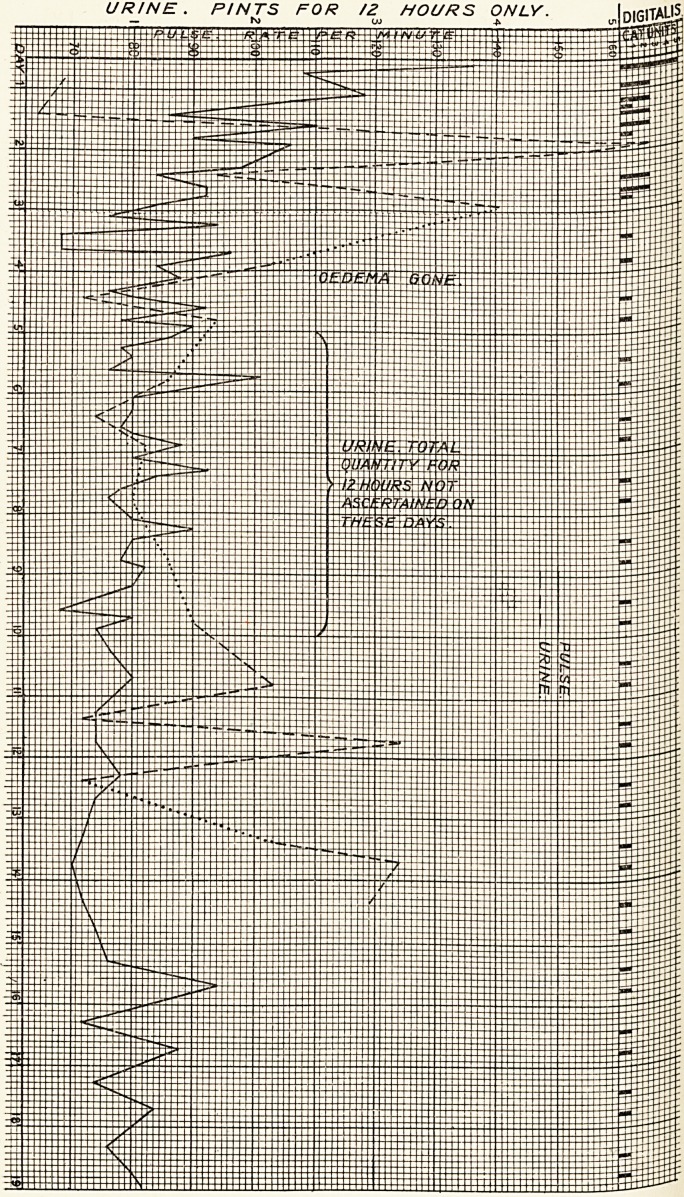# A Case of Mitral Stenosis with Auricular Fibrillation, Treated with Digitalis by the Cary Eggleston Method

**Published:** 1928

**Authors:** K. H. Pridie


### A CASE OF MITRAL STENOSIS WITH AURICULAR FIBRILLATION, TREATED WITH DIGITALIS BY THE CARY EGGLESTON METHOD.

BY

K. H. Pridie.

The Cary Eggleston method of administration of digitalis is based upon using digitalis standardised in cat-units. A cat-unit of digitalis is the weight of dry drug in milligrams required to kill one kilogram of cat when an aqueous infusion of it is slowly injected intravenously. The Cary Eggleston dosage, to produce complete digitalisation, finds it necessary to give one cat-unit of digitalis for every ten pounds of the patient's body weight. The process of digitalisation can be spread over several days. About two cat-units of digitalis are eliminated by the body every day, so that two cat-units a day must be added to the dose calculated on the body weight. When digitalisation is complete, a dose of two cat-units a day will maintain it.

**Case.**—M. M., female, age 62, admitted to Southmead Hospital on February 7th, 1928, complaining of swelling of legs, right arm, abdomen and eyelids, and great shortness of breath. The patient stated that she had had two similar attacks and heart trouble before, the first in 1925. At Christmas, 1927, she was in the Bristol Royal Infirmary, recovered and went home, and since then had taken no medicine. For the three weeks previous to admission she had been getting worse and was confined to bed. Her right arm began to swell the day before admission.

On admission the patient was very dyspnœic and cyanosed, her lips blue and the skin of the face and chest pigmented. The right arm was œdematous and pitted on pressure, and there was great œdema of both legs and flanks. The abdomen was distended and dull to percussion, especially in the flanks, the

URINE. PINTS FOR 12 HOURS ONLY.

PULSE. RATE PER MINUTE

DIGITALIS

CAT UNITS

DAY

OEDEMA GONE.

URINE. TOTAL QUANTITY FOR 12 HOURS NOT ASCERTAINED ON THESE DAYS.

PULSE.

URINE.

dullness being shifting. There was swelling of the lower eyelids. Temperature 97·48.

Ventricular rate 156, very irregular in rhythm and force. Area of cardiac dullness upper limit at upper border of third rib, apex beat at fifth space one inch exterior to mid-clavicular line, inner limit at right sternal border. First sound loud, banging, totally irregular in rate and strength. Second sound not heard. Loud systolic murmur heard over apex and conducted outwards into the axilla.

Respiratory rate 32. Patient showed marked cyanosis, was very dyspnœic and sat propped up in bed to breathe. Percussion showed dullness over both bases. On auscultaticn bubbling râles were heard over the chest. Urine very scanty, amber, 1026 s.g., acid albumen present.

It was determined to digitalise the patient, spreading the process over three days. The weight of the patient was 140 lbs., so that fourteen cat-units of digitalis were required, allowing one cat-unit for every ten pounds of body weight, with the addition of two cat-units per day for three days to replace that eliminated, making twenty cat-units in all. Ten cat-units were given in the first twenty-four hours and five each in the second and third. Thereafter one cat-unit was given morning and evening daily to maintain digitalisation. No drugs were given other than powdered digitalis leaf in tablets standardised in cat-units (Upsher Smith brand).

February 7th, 1928. Digitalisation started at 4.0 p.m. with five cat-units, followed by two cat-units at 10.0 p.m. Urine eight oz. night. Patient did not sleep.

February 8th, 1928. Two cat-units at 4.0 a.m., one cat-unit at 6.0 a.m. Urine four oz. day. Two cat-units at 10.0 a.m. two cat-units at 2 0 p.m., one cat-unit at 6.0 p.m. Urine, five pints two oz. night.

February 9th, 1928. Œdema reduced. Two cat-units at 10.0 a.m., two cat-units at 2.0 p.m., one cat-unit at 6.0 p.m. Digitalisation complete. Urine, thirty-four oz. day, four pints night.

February 10th, 1928. Dosage altered to one cat-unit every morning and evening to maintain digitalisation. Patient felt much better, œdema almost gone, no nausea, appetite good, slept well. Urine, two pints two oz. night.

February 11th, 1928. Pulse regular, 92 per min. Presystolic murmur heard at apex, also systolic murmur. Urine, twelve oz. night, thirty-four oz. day.

February 21st, 1928. Patient got up for half an hour. Pulse while up 72 per min.

February 28th, 1928. Patient much better, walking about. Pulse while up 88 per min.

The attached chart shows the heart rate, urine passed and dosage of digitalis.

### COMMENTS.

This case affords an illustration of the value of the Eggleston principle, that calculation of the dosage in terms of the patient's body weight makes it possible to bring about digitalisation rapidly and at the same time safely.

By this means early cardiac failure of the auricular type can be dealt with quickly, as quickly indeed as by injections of strophanthin, and at the same time more safely.

The fact that in this method the larger fractions of the dosage are given when the patient is free from digitalis, and that the dose is reduced as his tissues approach saturation, is a valuable source of protection to the patient, and one which usually makes it possible to avoid all evidences of digitalis intoxication while still securing the full benefits of the drug.

The subsequent treatment of the patient is rendered very simple. The patient takes from one to four cat units per day according to his rate of elimination of the drug.

The use of a preparation that has been physiologically standardised is necessary, so that the physician may be confident that his calculation of dosage in terms of body weight and physiological activity can be relied on.

I have to thank Dr. Phillips of Southmead Hospital for permission to publish this report.

## Figures and Tables

**Figure f1:**